# Effect of Different Body Postures on the Severity of Stuttering in Young Adults with Developmental Stuttering

**DOI:** 10.1155/2019/1817906

**Published:** 2019-08-05

**Authors:** Abdulaziz Almudhi, Hamayun Zafar, Shahnawaz Anwer, Ahmad Alghadir

**Affiliations:** ^1^Department of Medical Rehabilitation Sciences, College of Applied Medical Sciences, King Khalid University, Abha, Saudi Arabia; ^2^Department of Rehabilitation Sciences, College of Applied Medical Sciences, King Saud University, Riyadh, Saudi Arabia; ^3^Rehabilitation Research Chair, College of Applied Medical Sciences, King Saud University, Riyadh, Saudi Arabia; ^4^Department of Building and Real Estate, Hong Kong Polytechnic University, Kowloon, Hong Kong Special Administrative Region, China

## Abstract

**Objective:**

The current study aimed to investigate the effects of body position on the level and severity of stuttering in young adults with developmental stuttering.

**Methods:**

A total of 24 subjects (male: 17; female: 7; mean age: 24.9 ± 6.2 years) with developmental stuttering participated. The participants were asked to perform oral reading and spontaneous monologue-speaking tasks in different body postures while their speech was recorded. During reading and speaking tasks, the Stuttering Severity Instrument was used to quantify the severity of stuttering. The effects of different body postures on stuttering severity, reading task, and speaking task scores were analyzed.

**Results:**

Significant differences in stuttering severity, reading task, and speaking task scores were found for different body postures. Post hoc analyses revealed a significant difference in stuttering severity, reading task, and speaking task scores when subjects were sitting on a chair with no arm support compared to lying down (p<0.05). Similarly, there were significant differences for two sitting positions (sitting on a chair with no arm support vs sitting on a chair with arm support (p<0.05)).

**Conclusions:**

Body postures or body segment positions that relax and facilitate the muscles of the neck and shoulders may potentially improve speech fluency in young adults with developmental stuttering.

## 1. Introduction

Stuttering is a complex communication disorder with affective, behavioral, and cognitive components [1] and psychological and social effects [2]. There is a widespread agreement that between 4 and 5% of the preadolescent population is stutterers [3–5]. The prevalence of stuttering in the adult population may be less than 1% [6]. It has been estimated that the prevalence of stuttering in the global population is 1% at any time [3, 5, 7].

There are different treatment approaches for children and adults who stutter since strategies that may work well for preschool children may be of little use for adults [8]. Some therapeutic approaches and treatment programs may allow a person with stuttering (PWS) to improve speech fluency and communicate in an effective way [9]. Alternative approaches may instead focus on cognitive, behavioral, or psychological therapy or use prosthetic devices that deliver altered auditory feedback [10, 11]. Although researchers strive to understand the key factors associated with therapy success, there is limited information about the exact mechanisms underlying stuttering. Therefore, researchers and clinicians can formulate therapy goals based on the psychological differences between PWS and nonstutterers [12]. Long-term treatment studies can also provide insights into treatable and refractory stuttering behaviors [13]. These types of studies can provide a deeper understanding of stuttering [14, 15].

Previous studies had suggested that stuttering is fundamentally considered as a speech motor disorder in addition to language, psychological, or other issues causing further progress of fluency disruptions in both children and adults [16–18]. Past studies have investigated the role of proprioceptive feedback to control and coordinate speech movements [19, 20]. The results of their study demonstrated that the PWS showed less efficient use of proprioceptive feedback to control speech-related or nonspeech-related movements [19, 20]. For example, De Nil and Abbs [20] have indicated that PWS showed reduced use of proprioceptive feedback to control a very small amplitude lip, jaw, and tongue movements. Additionally, PWS showed inaccuracy in the movement control compared to nonstuttering individuals [20]. Moreover, the stuttering severity was significantly correlated with the ability to control even a small movement [19]. However, interestingly, patients with mild stuttering showed poorer use of proprioceptive feedback movements control compared to those with moderate or severe stuttering [21]. In addition, more severe stuttering patients showed significantly slower movements compared to mild or moderate stuttering [21]. Thus, they proposed that, when depending on proprioceptive feedback, patients with more severe stuttering are likely to reduce their movements in order to improve performance and accuracy [21].

Although normal and pathological (e.g., stuttering) speech production depends on multiple factors (i.e., physiological, psychological, neurological, genetic, and environmental factors), sensorimotor control of speech is of paramount importance [13]. Proprioceptive feedback plays a vital role in speech production [21]. The previous study has suggested that this feedback helps us to quickly and usually unknowingly compensate articulatory discoordination [22]. Proprioception is also important for temporospatial coordination of fluent articulatory movements [23]. Training of new motor skills requires the availability of fast and accurate proprioceptive function [24]. This afferent feedback information combined smoothly and quickly with efferent motor signals to help movement to achieve desired outcome [24]. Previous studies suggested that even fluent speech in PWS has been often discoordinated, indicating the presence of a more established motor deficit [25, 26]. It is currently known that body positioning and posture facilitate neck and shoulder muscles [27]. Moreover, the functional relationship between the neck and jaw has been described [28]. Thus, we suggest that any type of body posture or body segment positioning that facilitates or reduces the load on the neck or shoulder muscles has the potential to improve proprioceptive feedback and fluent articulatory movements in PWS. Therefore, the present study aimed to investigate the effects of body position on stuttering severity in young adults with developmental stuttering. We hypothesized that the lying down position followed by sitting on a chair with arms would be the most effective positions for reducing stuttering severity, as these positions provide maximal body relaxation, which improves speech production.

## 2. Methods 

### 2.1. Study Participants

A total of 24 subjects (male: 17; female: 7; mean age: 24.9 ± 6.2 years) participated in this study. The subjects had attended stuttering clinics prior to the start of this study. Participant inclusion criteria were as follows: (a) history of stuttering onset prior to six years of age [29]; (b) no motor development issues; (c) no concurrent language or speech development issues; (d) not taking medication that potentially affected articulation, depression, phonation, or respiration; (e) no diagnosis of psychiatric or neurological disorders, cranial nerve VII or hearing impairments, or epilepsy; (f) diagnosis of persistent developmental stuttering; and (g) no therapy of any kind during the previous 12-month period. Consultant Speech Language Pathologist with experience of 13 years made the diagnosis of stuttering in the current study.

### 2.2. Procedure

This study was conducted at King Saud University–Speech Clinics in Riyadh, Saudi Arabia. All subjects participated in therapy sessions. Subject speech samples were collected in an interview room under two conditions: (1) spontaneous speaking and (2) reading task. A camera and tape recorder were used for session recording.

#### 2.2.1. Stuttering Severity Instrument Measurements

The Stuttering Severity Instrument (SSI-4), 4th Edition [30], quantifies disfluency duration, frequency, and physical features in preschool children through adults. The SSI-4 enables the assessment of behavioral severity levels in readers and nonreaders. Classification of stuttering severity based on the total score and percentile ranks is as follows: very mild (total score, 10-17; percentile rank, 1-11), mild (total score, 18-24; percentile rank, 12-40), moderate (total score, 25-31; percentile rank, 41-77), severe (total score, 32-36; percentile rank, 78-95), and very severe (total score, 37-46; percentile rank, 96-99) [30]. Although there are many fluency assessment tools available, the SSI-4 is highly recommended due to its thoroughness and procedural reliability. A recent study indicated high interrater relative reliability and intrarater absolute reliability of SSI-4 [31]. For our data analysis, SSI-4 total severity, reading task, and speaking task scores were the dependent variables.

#### 2.2.2. Individual Assessment Session Methods

The participants were asked to perform oral reading and spontaneous monologue-speaking tasks in different body positions, which are described in Table 1. Testing condition order and reading and speaking tasks were randomized to minimize experimenter bias and adaptation effects, respectively. During these tasks, participants were recorded using a Sony IC Recorder (ICD-AX412F). During the speaking task, participants were told to speak for at least four minutes on any subject (e.g., work or leisure). Moreover, each participant was asked to speak approximately 300 words. To ensure clear recording, a 30-40 cm distance was maintained between the recording device and each participant. Each participant was asked to read an Arabic standardized passage. In each task, the average syllables must be 500.

### 2.3. Statistical Analysis

Statistical analysis was performed using SPSS 22.0 Software (IBM Inc., Chicago, IL, USA). Descriptive statistics (i.e., mean, standard deviation, and percentages) were reported for stuttering severity, reading task, and speaking task scores in different postural conditions. Normality of data was tested using the Shapiro-Wilk test (p>0.05). The effects of different postural conditions on stuttering severity, reading task, and speaking task scores were analyzed using analysis of variance (ANOVA) with repeated measures at a statistical significance level of 0.05. If interactions of different postural conditions were detected, a post hoc analysis with Bonferroni adjustment was employed.

## 3. Results

### 3.1. Participant Characteristics

Descriptive statistics for severity, reading task, and speaking task scores associated with different postural conditions are presented in Table 2. When subjects sat on a chair with no arms, the majority had moderate or severe stuttering severity scores. Most subjects had mild stuttering severity scores associated with lying on a bed. The majority of subjects had mild or moderate stuttering severity scores when standing. Nearly equal numbers of subjects had mild or moderate stuttering severity scores associated with sitting on a chair with arms. When subjects stretched their neck muscles, most had a moderate stuttering severity score.

### 3.2. Comparisons of Severity, Reading, and Speaking Task Scores Associated with Different Body Positions

Comparisons of severity, reading task, and speaking task scores associated with different postural conditions are presented in Table 3. There were significant differences between stuttering severity, reading task, and speaking task scores associated with different postural conditions. Post hoc analysis revealed significant differences in stuttering severity, reading task, and speaking task scores when subjects were sitting on a chair with no arms compared to lying on a bed (p < 0.05). Similarly, there were significant differences in these scores when subjects were sitting on a chair with no arms compared to a chair with arms (p < 0.05).

## 4. Discussion

The current study aimed to evaluate stuttering severity for five different postural conditions using the SSI-4. The results of the current study identified that lying down position was the most effective position for reducing stuttering severity. Most subjects in this position displayed mild stuttering severity. Sitting on a chair with arms was the second most effective position for reducing the severity of stuttering. In this position, half of the subjects displayed mild stuttering. None of the subjects showed severe or very severe stuttering while lying down or sitting on a chair with arms. Sitting on a chair with no arms or standing was the least effective position for reducing stuttering severity. Half of the subjects displayed severe or very severe stuttering while sitting on a chair with no arms. Moreover, while in a standing position, half of the subjects displayed moderate stuttering.

Compared to other postural conditions, lying down and sitting on a chair with arms had the lowest SSI-4 severity, reading task, and speaking task scores. Speech is considered to be the most complex motor activity, involving precise coordination of orofacial, laryngeal, and respiratory muscles. We suggest that the most effective body position (i.e., lying down and sitting on a chair with arms) supports maximal body relaxation, which might improve speech production. However, these relationships need to be investigated in more robust controlled studies in the future. It is further recommended to assess the impact of different body position on proprioceptive feedback during speech production to further validate these findings.

The muscles of the neck and jaw consist of densely populated muscle spindles [32], which can be activated by slight changes in the neck or body position. It was hypothesized that, similar to other motor functions, speech motor output depends on incoming proprioceptive and sensory information. Moreover, sensorimotor control plays a vital role in the production of speech [21]. Furthermore, Loucks et al. [33] indicate that PWS showed reduced proprioceptive function and poor oral motor task performance during specific target oriented jaw movements. Reduced proprioceptive measures have been associated with reduced ability to coordinate jaw movements with phonation, indicating the importance of this factor during articulatory discoordination often noticed in PWS [33]. The findings of the previous study indicate that people with severe stuttering who modify their movement strategy in order to improve movement accuracy may advocate that some of the noticed changes in the pattern of proprioceptive response could have resulted by movement strategy instead of sensory sensitivity [21]. Another study reported an association of increased movement accuracy and a slowing of movement speed, which indicates PWS become more fluent if they reduce their rate of speech. It is assumed that the impact of slowing speech on improved fluency could have been driven by an improved proprioceptive response [19]. Therefore, in the present study, we hypothesized that different body positions or postures may relax the muscles of the neck and upper extremities, which, in turn, may improve speech fluency in patients with developmental stuttering. During initial therapy sessions, using these body positions may help to correct speech motor output.

Based on our findings, we recommend that developmental stuttering therapy should begin with patients lying down during sessions since this position is the most comfortable for patients and reduces stuttering. While in this position, the patient's central nervous system may receive improved sensory inputs, leading to correct motor output. Once the level of stuttering decreases, patients can progressively experience different postural conditions during therapy sessions, such as sitting on a chair with arm support, standing, or sitting on a chair with no arm support.

The present study has several limitations. First, although our study only included subjects over 18 years old, we recognize that stuttering generally begins in childhood and more research is needed in this area. Second, although it is typical for the field, the small sample size (n = 24) of our study limits the validity of the results. Moreover, the use of mean scores may have masked the variations in some scores, for example, extreme high or low; however, due to the small sample size of our study, we cannot assess this. Finally, the validity of this study is limited given the lack of a control group.

## 5. Conclusions

Body postures or body segment positions that relax and facilitate the muscles of the neck and shoulders may potentially improve speech fluency in young adults with developmental stuttering. These findings may be of clinical significance; however, more longitudinal studies are needed to validate the results of this study.

## Figures and Tables

**Table 1:** Description of body positions during the assessment of the severity score, reading task score, and speaking task score.

No	Positions	Action
1	Chair with no arms	The clinician asks the client to sit on a chair with no arms and then the clinician asked the client to read a chosen article and speak spontaneously in a preferred subject. These 2 tasks are recorded for the evaluation and follow-up purposes.

2	Lying down	The clinician asks the client to lie down on a bed and then the clinician asked the client to read a chosen article and speak spontaneously in a preferred subject. These 2 tasks are recorded for the evaluation and follow-up purposes.

3	Standing	The clinician asks the client to stand on feet and then the clinician asked the client to read a chosen article and speak spontaneously in a preferred subject. These 2 tasks are recorded for the evaluation and follow-up purposes.

4	Chair with arms	The clinician asks the client to sit on a chair with arms and then the clinician asked the client to read a chosen article and speak spontaneously in a preferred subject. These 2 tasks are recorded for the evaluation and follow-up purposes.

5	Stretch neck muscles	The clinician asks the client to wrap the client's neck with neck stretcher and then the clinician asked the client to read a chosen article and speak spontaneously in a preferred subject. These 2 tasks are recorded for the evaluation and follow-up purposes.

Note: all the participants read the same passage of chosen article in all five conditions.

**Table 2:** Descriptive statistics of the severity score, reading task score, and speaking task score in different postural conditions.

Group	Severity score, grading (% of the participants)	Reading task score (% of the participants)	Speaking task score (% of the participants)
Chair with no arms	Mild (14.3)	2 (7.1)	3 (7.1)
	Moderate (35.5)	4 (7.1)	7 (21.4)
	Severe (35.6)	5 (14.3)	8 (21.4)
	Very severe (14.6)	6 (42.9)	9 (50)
		7 (7.1)	
		8 (7.1)	
		9 (14.3)	

Lying down	Mild (64.5)	2 (28.6)	2 (7.1)
	Moderate (35.5)	4 (35.7)	4 (14.3)
		5 (14.3)	5 (7.1)
		6 (14.3)	6 (42.9)
		7 (7.1)	7 (21.4)
			8 (7.1)

Standing	Mild (28.5)	2 (7.1)	3 (7.1)
	Moderate (49.8)	4 (21.4)	5 (7.1)
	Severe (21.7)	5 (28.6)	6 (14.3)
		6 (28.6)	7 (35.7)
		7 (14.3)	8 (21.4)
			9 (14.3)

Chair with arms	Mild (49.9)	2 (14.2)	2 (7.1)
	Moderate (50.1)	4 (42.9)	4 (7.1)
		5 (35.8)	5 (7.1)
		6 (7.1)	6 (42.9)
			7 (28.7)
			8 (7.1)

Stretch neck muscles	Mild (35.6)	2 (7.1)	3 (7.1)
	Moderate (64.4)	4 (28.6)	4 (7.1)
		5 (50)	6 (50)
		6 (14.3)	7 (21.4)
			8 (14.3)

**Table 3:** Comparisons of the severity score, reading task score, and speaking task score in different postural conditions.

	Chair with no arms	Lying down	Standing	Chair with arms	Stretch neck muscles	*∗*p-value	Significant difference between tested postural conditions	
							Chair with no arms vs Lie down	Chair with no arms vs Chair with arms
Severity score†	68.4 SD 26.8	42.9 SD 18.6	57.9 SD 20.6	44.5 SD 16.2	49.6 SD 15.5	.007	.012*∗*	.024*∗*
Reading task score†	6.1 SD 1.8	3.8 SD 1.9	5.1 SD 1.5	4.2 SD 1.2	4.6 SD 1.2	.004	.003*∗*	.028*∗*
Speaking task score†	7.9 SD 1.6	5.7 SD 1.5	6.9 SD 1.6	5.9 SD 1.5	6.1 SD 1.4	.002	.003*∗*	.009*∗*

*∗*Repeated-measure ANOVA test (p<0.05). Data are presented as mean with standard deviation.

†Severity, reading, and speaking task scores were given in percentile rank.

## Data Availability

All data generated or analyzed during this study are presented in the manuscript. Please contact the first author for access to data presented in this study.

## Abbreviations

PWS: People who stutter
EBM: Evidence-based medicine
EBP: Evidence-based practice
SSI: Stuttering Severity Instrument
SLP: Speech-language pathologists.
